# COVID-19 Infection and Symptoms Among Emergency Medicine Residents and Fellows in an Urban Academic Hospital Setting: Cross-sectional Questionnaire Study

**DOI:** 10.2196/29539

**Published:** 2022-01-27

**Authors:** Stacey Frisch, Sarah Jones, James Willis, Richard Sinert

**Affiliations:** 1 Department of Emergency Medicine SUNY Downstate Health Sciences University Brooklyn, NY United States; 2 Department of Emergency Medicine Jackson Health System Miami, FL United States

**Keywords:** COVID-19, emergency medicine, housestaff wellness, medical education, training, frontline health care workers, frontline, personal protective equipment, pandemic, infectious disease, emergency

## Abstract

**Background:**

COVID-19, an illness caused by the novel coronavirus SARS-CoV-2, affected many aspects of health care worldwide in 2020. From March to May 2020, New York City experienced a large surge of cases.

**Objective:**

The aim of this study is to characterize the prevalence of illness and symptoms experienced by residents and fellows in 2 New York City hospitals during the period of March to May 2020.

**Methods:**

An institutional review board–exempt survey was distributed to emergency medicine housestaff in May 2020, and submissions were accepted through August 2020.

**Results:**

Out of 104 residents and fellows, 64 responded to our survey (a 61.5% response rate). Out of 64 responders, 27 (42%) tested positive for SARS-CoV-2 antibodies. Most residents experienced symptoms that are consistent with COVID-19; however, few received polymerase chain reaction testing. Out of 27 housestaff with SARS-CoV-2 antibodies, 18 (67%) experienced fever and chills, compared with 8 out of 34 housestaff (24%) without SARS-CoV-2 antibodies. Of the 27 housestaff with SARS-CoV-2 antibodies, 19 (70%) experienced loss of taste and smell, compared with 2 out of 34 housestaff (6%) without SARS-CoV-2 antibodies. Both fever and chills and loss of taste and smell were significantly more commonly experienced by antibody-positive compared to antibody-negative housestaff (*P*=.002 and <.001, respectively). All 13 housestaff who reported no symptoms during the study period tested negative for SARS-CoV-2 antibodies.

**Conclusions:**

Our study demonstrated that in our hospitals, the rate of COVID-19 illness among emergency department housestaff was much higher than previously reported. Further studies are needed to characterize illness among medical staff in emergency departments across the nation. The high infection rate among emergency medicine trainees stresses the importance of supplying adequate personal protective equipment for health care professionals.

## Introduction

COVID-19 is a viral respiratory illness caused by SARS-CoV-2; it has created problems worldwide since 2020. By March 2020, COVID-19, also known as “novel coronavirus,” had reached the epidemiological criteria for a global pandemic [1]. After its initial identification in Wuhan, China, COVID-19 quickly spread across the world [2]. Since COVID-19 was first identified in the United States on January 15, 2020, in Seattle, Washington, the United States has reported the largest number of confirmed cases. To date, the United States has had over 13.8 million COVID-19 cases, with over 320,000 of those in New York City alone [3]. New York City experienced a massive surge of cases between March and May 2020.

At the time of the writing of this article, the county of Kings, New York, also known as the city of Brooklyn, had seen the highest number of COVID-19–related deaths in the United States, at over 24,000 [2]. The State University of New York (SUNY) Downstate Medical Center and Kings County Hospital are state and public city hospitals located in central Brooklyn. The emergency departments in both hospitals are staffed primarily by board-certified emergency medicine (EM) attending physicians and EM residents. As of November 18, 2020, Kings County Hospital had cared for 2701 patients with COVID-19 and reported 348 COVID-19–related deaths. As of November 18, 2020, SUNY Downstate Medical Center, which was designated a COVID-19–only facility by the state governor’s mandate [4], had admitted 864 patients with COVID-19 and reported 298 deaths.

Resident physicians in teaching hospitals act as the front lines of the emergency department, intensive care units, and clinics. Given the large volumes of patients they see over long and frequent shifts, their exposure rates are perceived to be great. Furthermore, in this study, we include SARS-CoV-2 exposure early in the first wave of COVID-19, when personal protective equipment (PPE) was limited and before stockpiles were mandated in New York City.

This study aims to evaluate the SARS-CoV-2 exposure of emergency medicine resident physicians and fellows working at the abovementioned urban academic medical centers. After quantifying the exposure, their symptoms, the number of patients with COVID-19 treated and intubated, and perceived adequacy of PPE was correlated with residents’ and fellows’ antibody test results.

## Methods

### Study Design, Setting, and Population

A cross-sectional survey study was conducted at SUNY Downstate Medical Center and Kings County Hospital Center in Brooklyn, New York, among individual emergency medicine residents and pediatric emergency medicine fellows. This material has not been previously presented.

### Study Protocol

The open 20-question electronic survey questionnaire was generated using the Qualtrics Survey platform, August 2020 version (Qualtrics), and the technical functionality of the survey on the Qualtrics platform was tested prior to distribution. The survey was self-administered in May 2020 via email listserv to the residents and fellows of the SUNY Downstate Emergency Medicine Department. The survey and investigation received institutional review board (IRB) exemption status from the SUNY Downstate IRB with participant consent waived. Participation in the study was voluntary, and no compensation was given for participation. No personal information was stored. Completeness checks were not performed automatically, but participants were able to review their responses prior to submitting. Results were automatically captured in the Qualtrics system, and they were kept anonymous and confidential. IP addresses were used to ensure unique responses and identify potential duplicate entries. During the study period, residents were offered three laboratory options for SARS-CoV-2 IgG antibody testing:

1. Wadsworth Center microsphere immunoassay [5], performed at the public health laboratory of the New York State Department of Health
2. Abbott Laboratories Inc chemiluminescent microparticle immunoassay [6], performed at Quest Diagnostics
3. Abbott ARCHITECT [6] nucleocapsid immunoassay analyzer, performed at the University Hospital of Brooklyn Laboratory

Residents who had reverse transcriptase–polymerase chain reaction (RT-PCR) testing were offered the following tests from our institutions:

1. Hologic Panther Fusion System [7], performed at Lenco Diagnostic Laboratory (March 2020)
2. Cepheid GeneXpert Systems [8], conducted at the University Hospital of Brooklyn Laboratory (April to August 2020)
3. BioFire Respiratory 2.1-EZ Panel [9], conducted at the University Hospital of Brooklyn Laboratory (July to August 2020)

### Key Outcome Measures

The survey questions included a range of options for the total number of patients with COVID-19 that the housestaff were exposed to, the total number of patients with COVID-19 that the housestaff intubated, average clinical weekly hours worked, symptoms of illness, and whether or not the housestaff felt the PPE provided was adequate. The survey questions referenced the period between February 2020 and survey completion. Results were collected through August 2020.

### Data Analysis

Survey responses were tabulated and compiled in table format with ranges. Frequency data were reported as percentages with 95% confidence intervals. The Fisher exact test was used to analyze group comparisons. The α value was set as .05; all tests were 2-tailed (SPSS, version 23.0; IBM Corporation).

## Results

The demographic characteristics of the survey participants are presented in Table 1.

**Table 1:** Demographics of survey participants (N=64).

Characteristics		Value, n (%)
**Age (years)**		
	26-30	39 (61)
	31-35	21 (33)
	36-40	4 (6)
**Postgraduate year**		
	1	13 (20)
	2	20 (31)
	3	14 (22)
	4	12 (19)
	5+	5 (8)
**Gender**		
	Female	33 (52)
	Male	31 (48)
**Clinical hours** **(average/week)**		
	11-20	1 (2)
	21-30	4 (6)
	31-40	11 (17)
	41-50	17 (27)
	51-60	23 (36)
	61-70	6 (9)
	71-80	2 (3)
**COVID-19 PCR^a^ test result**		
	Positive	9 (14)
	Negative	8 (12)
	Indeterminate	1 (16)
	Did not take PCR test	46 (72)
**Antibody test result**		
	Positive	27 (42)
	Negative	34 (53)
	Indeterminate	1 (16)
	Did not take antibody test	2 (3)

^a^PCR: polymerase chain reaction.

Of 104 residents and fellows, 64 responded to the survey, yielding a 61.5% response rate. There were no duplicate entries, and all surveys were filled out completely. Of the 64 housestaff, 27 (42%) tested positive for SARS-CoV-2 IgG antibodies, 2 residents did not undergo antibody testing, and 1 resident had indeterminate results. Most of the respondents were female (33/64, 52%) and between 26 and 30 years of age (39/64, 61%). The most common postgraduate year (PGY) was PGY2, with PGY3 and PGY1 the second and third most common, respectively. Most of the housestaff (23/64, 36%) worked 51 to 60 hours per week. The majority of study participants (46/64, 72%) did not take a SARS-CoV-2 PCR test, but 62 of 64 (97%) of respondents had taken a SARS-CoV-2 antibody test. All residents with a positive PCR test (n=9) also had a positive antibody test.

Table 2 compares COVID-19 exposure between residents who tested antibody-positive and antibody-negative for SARS-CoV-2. There was no significant difference in the risk of a positive antibody test based on the number of patients with COVID-19 the respondents treated during the study period. Most respondents (46/61, 75%) intubated fewer than 5 patients with COVID-19 at the time of the survey; this number of events is too small to accurately compare the number of intubations to the risks of becoming antibody-positive. A significant difference in symptoms was noted between antibody-positive and antibody-negative residents. Although none of the antibody-positive residents had no symptoms, 21 of 34 (62%) of the antibody-negative residents had a symptom commonly associated with COVD-19 infection.

Out of 27 housestaff with SARS-CoV-2 antibodies, 18 (67%) experienced fever and chills, compared with only 8 out of 34 housestaff (24%) without SARS-CoV-2 antibodies. A total of 19 out of 27 (70%) housestaff with SARS-CoV-2 antibodies experienced loss of taste and smell, compared with only 2 out of 34 (6%) housestaff without SARS-CoV-2 antibodies. Both fever and chills and loss of taste and smell were significantly more commonly experienced by antibody-positive compared to antibody-negative housestaff (*P* values .002 and <.001, respectively). Gastrointestinal and upper respiratory symptoms and headache did not appear to correlate to antibody status. The perception of the adequacy of PPE was similar regardless of antibody status.

**Table 2:** Comparison of the characteristics of COVID-19 IgG antibody-negative and antibody-positive residents.

Category		Antibody-negative (n=34), n (%)		Antibody-positive (n=27), n (%)		*P* value
**Patients treated, n**						
	<10	1 (3)	1 (4)		>.99	
	10-20	0 (0)	1 (4)		>.99	
	20-40	8 (24)	4 (15)		.52	
	40-60	8 (24)	7 (26)		>.99	
	60-80	6 (18)	5 (19)		>.99	
	80-100	2 (6)	1 (4)		>.99	
	>100	9 (26)	8 (30)		>.99	
**Patients intubated, n**						
	<5	26 (76)	20 (74)		>.99	
	5-10	2 (6)	6 (22)		*.049* ^a^	
	10-15	3 (9)	1 (4)		*.02*	
	15-20	3 (9)	0 (0)		.25	
**Resident symptoms of illness**						
	Fever and chills	8 (24)	18 (67)		**.** *002*	
	Gastrointestinal symptoms	8 (24)	8 (30)		.77	
	Upper respiratory symptoms	13 (38)	15 (56)		.21	
	Loss of taste/smell	2 (6)	19 (70)		*<.001*	
	Headache	11 (32)	11 (41)		.40	
	None	13 (38)	0 (0)		*<.001*	
**Adequate personal protective equipment**						
	Yes	18 (52)	11 (41)		.44	
	Maybe	10 (29)	9 (33)		.79	
	No	6 (18)	7 (26)		.25	

^a^Italic text indicates *P*<.05.

## Discussion

### Principal Findings

Our survey had an adequate response rate of 61.5% (64/104). Overall, 27 of 64 (42%) of our residents and fellows tested positive for SARS-CoV-2 antibodies, indicating a high exposure rate within the first few months of the pandemic. No residents or fellows were hospitalized. In residents who had SARS-CoV-2 antibodies, the most common symptoms experienced during the study period were loss of smell and taste (19/27, 70%), fever and chills (18/27, 67%), and upper respiratory symptoms (15 out of 27, 56%).

Sabetian et al [10] found a SARS-CoV-2 infection rate of 5.62% among 4854 health care workers in Southwest Iran between March and May 2020. They found that the highest infection rate was in emergency room workers (30.6%), which is comparable to our 42% infection rate for housestaff. Breazzano et al [11] surveyed cross-specialty program directors in New York City in April 2020, accounting for 382 emergency medicine residents; they found 6.5% confirmed, 8.4% presumed, and 3.1% suspected COVID-19 infections. These rates are much lower than the 42% infection rate of housestaff in our study because our study period extended through a longer time period, which allowed for more exposure and the availability of more testing in New York City. A more recent study in the US state of California, conducted from September to October 2020, found that only 2.9% of their emergency department staff (n=139) had antibodies for SARS-CoV-2 [12]. This study reported a much lower infection rate than ours, possibly because it was conducted before the largest surge of COVID-19 in California. Additionally, the New York City COVID-19 surge was the first large surge in our country, and the hospitals under study were unprepared, with insufficient PPE. By the time the California study was conducted, hospital workers were wearing N-95 masks universally. Lumley et al [13] investigated health care workers in the United Kingdom, and they found that 1265 out of 12,541 health care workers (10%) had SARS-CoV-2 antibodies by November 30, 2020. Their antibody prevalence was much lower than our 42% antibody prevalence, possibly because their study included health care workers who may have had fewer patient contact hours, such as administrative staff and laboratory staff; moreover, their study period concluded before the United Kingdom’s largest COVID-19 spike.

The percentage of physicians in training in our emergency departments who developed SARS-CoV-2 antibodies was greater than those previously reported. This is likely multifaceted and could be due to the high-risk nature of the EM specialty, the use of antibody testing in addition to PCR testing to determine exposure, location regulations, and our hospital and regional setting. Antibody testing captures the incidence of infections over a longer time frame (both active and past infections) compared to PCR testing, which usually only affords a positive result for an active infection. Additionally, our practice area of Flatbush, Brooklyn, was a COVID-19 hotspot, and the University Hospital of Brooklyn was identified as a COVID-19–only facility by governor mandate [4], which may have increased housestaff exposure.

Shahriarirad et al [14] investigated symptoms experienced by patients in Iran with COVID-19 and found that the most common symptoms at the onset of disease were fatigue (66.4%), cough (64.6%), and fever (59.3%). In our study, residents with SARS-CoV-2 antibodies had comparable rates of fever and chills (67%) and upper respiratory symptoms (56%). Additionally, the most common symptom experienced in our study was the loss of smell and taste (70%).

Alasia et al [15] found that advanced age and presence of fever, dry cough, dyspnea, fatigue, productive cough, diarrhea, and vomiting were more associated with severe COVID-19 disease among Nigerians in Rivers State. Our cohort did not have any cases of severe COVID-19 illness requiring hospitalization during the study period, and this may be because our cohort is composed of individuals aged 40 years and under.

Our study was not powered to detect a relationship between the number of patients seen and/or intubated and antibody status. A larger study is needed to evaluate this further. Another component that could be included in a further study is controlling for outside sick contacts, ensuring that the risk assessed for infection was work-related. Identification of exposure can be difficult, especially if the antibody test is used as a proxy for infection due to the longer time frame for positivity. Additionally, future studies should include vaccination status as a confounding variable for infection.

Lack of PPE at the onset of the pandemic was an issue nationally. More than half of our polled housestaff who developed SARS-CoV-2 antibodies stated that they felt the PPE provided to them may have been inadequate.

Only 18 of 64 housestaff (28%) had taken a SARS-CoV-2 PCR test when they answered the survey, although most of them reported symptoms. In comparison, 62 of 64 respondents (97%) reported having an antibody test within the same time frame. PCR testing identifies individuals with acute infection and active viral shedding and is also used to determine isolation needs. Our low reported PCR testing rate is likely due to the poor availability of PCR testing at the onset of the pandemic and could have contributed to asymptomatic spread of infection. PCR testing was limited to critically ill and hospitalized patients despite the presence of COVID-19–like symptoms.

The majority of housestaff, both those with and without antibodies, had symptoms consistent with COVID-19 during the study period. Fever and chills could be considered good symptoms for use in screening, but interestingly, only 66% of those who developed antibodies experienced fever or chills. Therefore, symptoms alone are not sufficient as a screening test. Loss of smell and taste was very specific in identifying individuals with SARS-CoV-2 antibodies. More data are needed to determine if other symptoms are sensitive and specific to identify COVID-19 illness in housestaff. These data are in line with multiple studies showing high false negative rates of SARS-CoV-2 PCR testing and stressing the use of a clinically based COVID-19 diagnosis [16-18]. The results of this study reinforce the accuracy of symptom-based diagnosis. Asymptomatic pooled PCR testing is another adjunct that can be used to identify individuals shedding viruses.

Our study is hypothesis-generating, and we would like to expand the survey across other emergency departments to gather more data. Because our study demonstrated a much larger percentage of residents experiencing COVID-19 illness compared to prior studies, we believe a larger study across multiple institutions and cities would be the next step in documenting housestaff illness and identifying causative factors, some of which may be possible to address during future waves of COVID-19 or other diseases with a similar spread.

Our study allows for both selection bias and recall bias. The 62% of housestaff who responded to the survey voluntarily may have been more skewed towards individuals who underwent antibody testing and had a particular result. Additionally, the survey retrospectively asked about the adequacy of PPE, and residents who tested positive for antibodies may have felt that due to their illness, they lacked PPE compared to their counterparts. Similarly, when asked retrospectively about symptoms of disease, our housestaff may have over- or underreported their symptoms.

Another limitation of our study was the relatively small sample size. Our study only included residents and fellows in 2 emergency departments in Brooklyn and therefore was underpowered to identify a significant trend in comparing patient encounters and intubations with COVID-19 illness in housestaff.

### Conclusion

The rate of COVID-19 infection in EM residents and fellows at 2 New York City hospitals during the first few months of the 2020 pandemic was 42%, much higher than that in previous reports. Other significant results include the association of fever and chills and loss of smell and taste with COVID-19 infection and the association of absence of any symptoms with SARS-CoV-2 antibody negativity in housestaff. This calls for continued advocacy for sufficient PPE and more routine PCR testing of asymptomatic carriers to identify those who are acutely ill and shedding virus.

## Abbreviations

EM: emergency medicine
IRB: institutional review board
PGY: postgraduate year
PPE: personal protective equipment
RT-PCR: reverse transcriptase–polymerase chain reaction
SUNY: State University of New York
